# Phylogenetic classifications are informative, stable, and pragmatic: the case for monophyletic taxa

**DOI:** 10.1007/s00040-016-0516-9

**Published:** 2016-09-19

**Authors:** P. S. Ward, S. G. Brady, B. L. Fisher, T. R. Schultz

**Affiliations:** 1 Department of Entomology and Nematology, University of California, Davis, CA USA; 2 Department of Entomology, Smithsonian Institution, Washington, DC USA; 3 Department of Entomology, California Academy of Sciences, San Francisco, CA USA

In a recent commentary in this journal Seifert et al. (2016) propose returning to a school of classification largely abandoned by systematists, in which both monophyletic and paraphyletic groups are formally recognized. This approach, dubbed “evolutionary classification”, has proved to be unattractive and impractical because the basis for taxon recognition is a confounding mix of phylogenetic relatedness and some measure of “degree of divergence”. Most systematists and evolutionary biologists now advocate classifications that are strictly phylogenetic, in which all named taxa above the species level are monophyletic (Wiley and Lieberman 2011; Vences et al. 2013; Judd et al. 2016). Hence, in contemporary biology textbooks birds are acknowledged to be part of the reptile clade; non-monophyletic groups such as “Pisces” and “Articulata” have been abandoned; and a primary division of flowering plants into “dicots” and monocots is recognized as untenable (Westheide and Rieger 2013, 2015; Freeman et al. 2014; Sadava et al. 2014; Judd et al. 2016). In entomology, paraphyletic groups such as “Homoptera”, “Heterocera”, and “Apterygota” are no longer part of insect classification (Gullan and Cranston 2014; Beutel et al. 2014). Others, such as Blattodea, have been redefined to encompass all their descendant taxa, and hence avoid paraphyly—in this case by including termites in the cockroach order (Inward et al. 2007). Of course, vernacular terms exist for some paraphyletic assemblages (moths, algae, fish, invertebrates, etc.), but most of them are no longer treated as formal groups in a classification.

One could argue that scientific controversies should not be decided by majority rule alone, but there are sound reasons why biological systematists overwhelmingly favor a phylogenetic classification. Such a scheme is simply more informative, accurate, and predictive. Birds really are a kind of modified reptile; to place them in a different group, separate from reptiles, obscures this important fact. Similarly, inclusion of termites in the order Blattodea emphasizes that they are indeed “social cockroaches” and this leads to a more insightful understanding of their biology and evolution (Bell et al. 2007). Excluding termites from Blattodea and putting them in their own order, Isoptera, would be positively misleading.

A phylogenetic classification is also, ultimately, more stable: as we refine our understanding of the tree of life, and achieve ever more confident estimates of phylogenetic relationships, systematists are more likely to converge upon a consensus. In a phylogenetic classification not all nodes in the tree of life need to be named, but any group that is named must meet the criterion of monophyly, and this limits the number of available options (Schmidt-Lebuhn 2012). By contrast, allowing paraphyletic groups opens up a can of worms. How distinct does a divergent ingroup have to be to justify excising it from its containing group and thereby render the latter paraphyletic? Given that rates of evolution are highly variable, and also vary among different classes of characters, there would be no end of argument—never resolved satisfactorily—about whether a given group is “sufficiently distinct” to be removed from its containing clade.

Consider the examples to which Seifert et al. (2016) objected. In our recent reclassification of the ant subfamily Myrmicinae (Ward et al. 2015), we placed lineages of socially parasitic ants into the genera in which they are embedded phylogenetically. Although these parasites had been placed in their own genera on the basis of their divergent phenotypes, our molecular phylogenetic results demonstrated that they are nested within more inclusive genera such as *Tetramorium* and *Temnothorax*, which each contain hundreds of species. Ongoing molecular studies show that the social parasites are situated shallowly within their respective host genera (F. Hita Garcia, pers. comm.; M. Prebus, pers. comm.), precluding a simple splitting into several monophyletic subgroups.

**Fig. 1:**
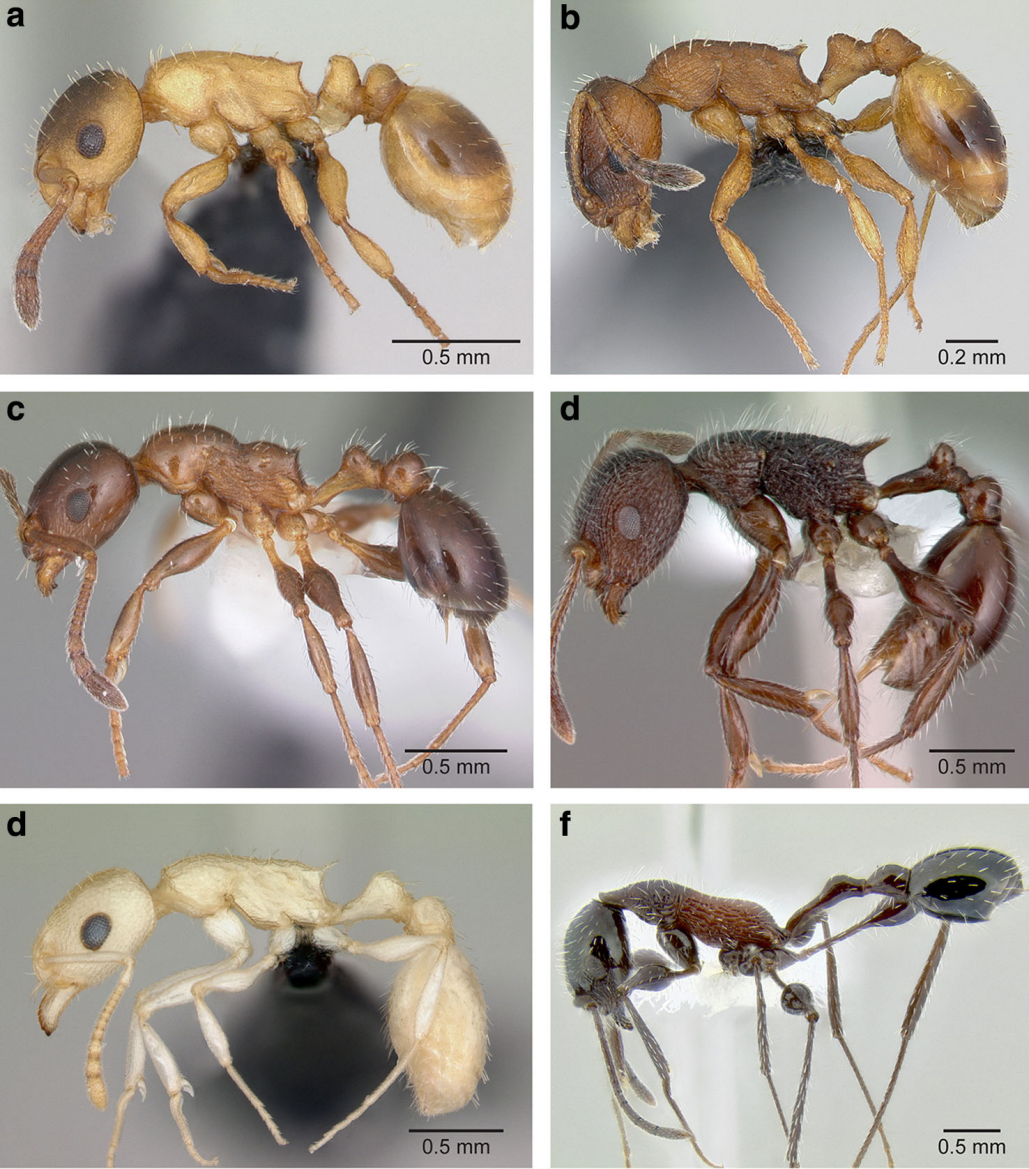
Morphological diversity in workers of the ant genus *Temnothorax*. **a** *T. ravouxi* (CASENT0173641), a social parasite formerly known as *Myrmoxenus ravouxi*, **b** *T. unifasciatus* (CASENT0173188), the most commonly used host species of *T. ravouxi*, **c** *T. pergandei* (CASENT0104016), formerly in subgenus *Dichothorax*, **d** *T. salvini* (CASENT0010847), part of a Mesoamerican radiation of the genus, **e** *T.* bca05 (CASENT0118165), a member of a species complex occurring in the deserts of Baja California, **f** *T. poeyi* (CASENT0106241), an extreme representative of the Caribbean radiation of *Temnothorax*. Images from AntWeb (http://www.antweb.org)

Moreover, contrary to the claim by Seifert et al. (2016) that the social parasites have diverged markedly while other congeners have remained more or less phenotypically static, there is a broad range of variability among the other species. For example, *Temnothorax* ants have undergone an impressive radiation in the Caribbean, producing species that are, at least superficially, far more divergent morphologically from typical Holarctic species of *Temnothorax* than the social parasites (Fig. 1). But there are varying degrees of extremeness in these Antillean *Temnothorax* (Fontenla 2000). Where along this range of variation should a break be made? Then there is the erstwhile subgenus *Dichothorax*, also well embedded in *Temnothorax*, with unusual mesosomal morphology. Should it be removed too? What about the pale nocturnal *Temnothorax* that have diversified in the deserts of Baja California? Or the Mesoamerican radiation of the *Temnothorax salvini* group? Depending on the whim and subjective perceptions of different “evolutionary systematists”, various parts of *Temnothorax* could be amputated, leaving behind an ill-defined assortment of species, scattered across the phylogeny.

No matter how such an operation is performed, it would always result in a loss of information content for *Temnothorax*. Under a phylogenetic classification, *Temnothorax* contains all members that share a most recent common ancestor. Under any paraphyletic formulation, this does not hold true. The increased efficiency of information retrieval that a phylogenetic system produces has been recognized in the literature for some time (Cracraft 1974; Farris 1979; see also Schmidt-Lebuhn 2013).

The argument that communication is hindered by adoption of a phylogenetic classification also does not stand up to scrutiny. The former parasite genera can be referred to using informal species-group names. For a period of time one could append the old genus name, e.g., “*ravouxi*-group (former *Myrmoxenus*)”, until usage of the species-group name takes over. There are numerous examples among ants of other satellite genera that were previously synonymized under their containing clades: *Doronomyrmex* under *Leptothorax*; *Sifolinia* under *Myrmica*; *Anergatides*, *Bruchomyrma*, *Sympheidole* and others under *Pheidole*; various former genera under *Strumigenys*, etc. No-one is any longer decrying the loss of these genus names; the species names (or informal species-group names) are still available and permit ready communication about the taxa concerned.

It is ironic that Seifert et al. (2016) exhort the reader to consider the experience of plant systematists. In fact, there have been major advances in the systematics of flowering plants, as botanists have developed a revised phylogenetic classification that incorporates the findings from molecular studies (Stevens 2016). The Angiosperm Phylogeny Group (APG) project is an excellent example of how the Linnaean system can be modified to accommodate new phylogenetic knowledge and to reflect relatedness (Angiosperm Phylogeny Group 2016). Seifert et al. (2016) fail to mention the APG initiative; instead they cite two botanists whose views (e.g., Stuessy & Hörandl 2014) are at variance with those of most plant systematists (cf. Kadereit et al. 2016).

Of course there can be challenges to the establishment of a ranked phylogenetic classification—in principle, for example, when dealing with putatively ancestral taxa (Schmidt-Lebuhn 2012), and in practice when faced with variable rates of morphological evolution among extant species (Ward 2011), and differing opinions about taxon circumscription and diagnosability (Christenhusz et al. 2015). The APG classification has undergone multiple iterations and is still the subject of ongoing discussion—motivated in part by new phylogenetic information, or by lingering uncertainties about relationships—but thanks to the unifying principle of phylogenetic relatedness a measure of consensus has been achieved that is difficult to imagine under the alternative of an “evolutionary classification”.

We are at an exciting time in the study of ants and other social insects where molecular data—increasingly at the genome scale (e.g., Blaimer et al. 2015)—is yielding unprecedented insight into their evolutionary history. Indeed, the whole-genome scans of socially parasitic ants and their hosts advocated by Seifert et al. (2016) have already commenced (e.g., in *Pogonomyrmex* and *Vollenhovia*; Smith et al. 2015), guided by the same phylogenetic context that also supports the classification of these social parasites in the same genera as their hosts. The advent of robust molecular phylogenies presents an opportunity to revise the taxonomy of social insects in line with new findings, and thereby establish a more stable and informative classification. The call by Seifert et al. (2016) to return to an outdated classification scheme would reverse this progress, and it should not be heeded.
